# Fatal sickling-associated microvascular occlusive crisis in a young with sickle cell trait

**DOI:** 10.4322/acr.2021.297

**Published:** 2021-08-20

**Authors:** Deepti Mutreja, Benjith Paul K, Tilak TVSVGK

**Affiliations:** 1 Armed Forces Medical College, Department of Pathology, Pune, Maharashtra, India; 2 Armed Forces Medical College, Department of Medicine, Pune, Maharashtra, India; 3 Command Hospital Air Force, Department of Laboratory Medicine, Bangalore, Karnataka, India

**Keywords:** Sickle Cell Trait, Autopsy, Military Personnel, Physical Exertion

## Abstract

Sickle cell trait (SCT), a heterozygous state characterized by hemoglobin AS, occurs commonly in sub-Saharan Africa, South America, Central America, India, and the Mediterranean countries. SCT is compatible with a normal lifespan and is not commonly regarded as a cause of morbid illness or death compared to its homozygous counterpart. We describe a case of fatal sickling-associated microvascular crisis, identified on post mortem evaluation in a previously undiagnosed 21-year-old military recruit with sickle cell trait. The individual presented with repeated syncope episodes during his training and was autopsied in the pursuit of cardiac anomalies and heat syncope. During the terminal episode, he collapsed and died of severe metabolic complications as he struggled to complete an organized run during routine training activities. To our knowledge, this is the first report of fatal sickling-associated crisis in a military recruit with sickle cell trait from India. This case serves to remind all armed forces and sports physicians of the importance of screening a recruit who is unable to complete exertional physical training for the presence of sickle cell trait.

## INTRODUCTION

The normal hemoglobin (Hb) in adults is composed of HbA and HbA_2_ with small quantities of HbF. Hemoglobin S (HbS), a structural hemoglobinopathy, in the heterozygous state, known as sickle cell trait (SCT), represented as HbAS, it is marked by the presence of 30-40% of HbS and the remainder being normal HbA.^1^ This hemoglobinopathy is fairly common in sub-Saharan Africa, South and Central America, , India, and middle eastern Arab countries, and the Mediterranean countries.^2^ In India, its prevalence ranges from 1-40% in certain belts.^3^^,^ 4

Although considered a benign hematological condition compatible with a normal lifespan, sickling-associated fatal complications in an SCT have rarely been described in the African-American recruits and athletes associated with severe physical exertion.^5^^-^ 7 Additional causes such as dehydration, heat stress, viral illness, and poor physical training have been presumed to play a role.^8^ In this report, we presented the post mortem findings of a young military recruit, an undiagnosed case of SCT, who died of fatal sickling-associated microvascular complications following an organized run during training activities. To our knowledge, this is the first documented report of this kind from the Indian subcontinent.

## CASE REPORT

A 20-year-old recruit, resident of north coastal Andhra Pradesh, a known sickle cell belt of India, was brought to the emergency with an alleged history of collapse and sudden loss of consciousness after a 2.2-km organized run. There was a history of non-recordable blood pressure and pulse, following which the medical officer proceeded with resuscitation maneuvers. On admission, the patient was unresponsive, hyperventilating without spontaneously opening his eyes or responding to pain. Pupils were bilaterally equal with sluggish response to light. Pulse was 100/min, Blood pressure was 110/60 mmHg, and respiratory rate was 42 breaths per minute.

Systemic examination revealed normal cardiovascular and chest findings. An arterial blood gas analysis showed a marked anion gap metabolic acidosis (pH 6.61, lactate 24.59, anion gap 30.4mmol/L, HCO3 4 mmol/L, pCO2 41.4 mmHg, sPO2 94%). Random blood sugar was 246 mg/dL. Electrocardiogram (ECG) showed features of hyperkalemia. The patient remained disoriented and started presenting a tense tender abdomen and decreased bowel sounds with dilated stomach, with distended bowel loops at the plain abdominal X-Ray. Despite fluid resuscitation, he continued to be anuric. The abdominal contrast-enhanced computed tomogram showed acute renal cortical necrosis with and distended bowels. A bedside 2D echogram was normal. Investigations revealed normal hemoglobin level, elevated total leukocyte counts (peripheral smear was characterized by normocytic normochromic red cells and neutrophil leukocytosis), deranged coagulation profile, hyperkalemia, markedly elevated liver enzymes, lactate dehydrogenase, and creatine kinase (Table 1). Blood cultures failed to detect bacterial growth.

**Table 1:** Laboratory work up

Exam	RR	PV H	CH		Exam	RR	PV H	C H	
			0900h	1430h				0900h	1430h
Hb	13.5-16.5g/dL	14.5	12.5	10.7	AST	<40UI/L	75	108	2434
Leukocyte	4000-11000/mL	13600	27400	5700	ALT	<40 UI/L	98	127	1295
Platelets	150-400x10^3^/mL	327	150	170	TP	6-8g/dL		7.2	5.2
BUN	7-23mg/dL	10	15	25	Albumin	3.5-4.8g/dL		4.4	3.3
Creatinine	0.6-1.3mg/dL	1.3	0.6	2.1	LDH	110-240 IU/L	-	250	668
Sodium	135-145 mmol/L	141	144	135	INR	1	-	1.68	1,85
Potassium	3.5-5.3mmol/L	5.0	6.8	8.3	PATT (R)	1	-	1,6	1.8
Chloride	97-110 mmol/L	106	103	108	CPK	30-300 IU/L	-	27	1504
TB	0.3-1.2 mg/dL	1.1	1.1	1.4	CKMB	5-25 IU/L			154
DB	0-0.4 mg/mL	0.4	0.6	0.7					

ALT= alanine aminotransferase; AST= aspartate aminotransferase; BUN= blood urea nitrogen; CH= current hospitalization; CK= creatine kinase; CKMB= creatine phosphokinase heterodimer MB; DB= direct bilirubin; Hb=hemoglobin; INR= international normalized ratio; LDH= lactate dehydrogenase; PPV H= previous hospitalization (one month prior); RR= reference range; TB= total bilirubin; TP=total protein; PATT= partial activated thromboplastin time.

The patient was managed with continuous renal replacement therapy. However, he desaturated, had a cardiac arrest, and died 18 hours after admission.

On perusal of medical documents of the patient, it was found that, in the past, there had been a history of two similar episodes. The patient had presented with presyncope following organized battle preparedness efficiency test (BPET) as a part of military training. He had been hospitalized following one such episode during which tall T waves on ECG and hyperkalemia had been documented. However, his cardiac evaluation, including electrocardiogram, Holter, and treadmill test, had been normal.

## AUTOPSY FINDINGS

**Figure 1:**
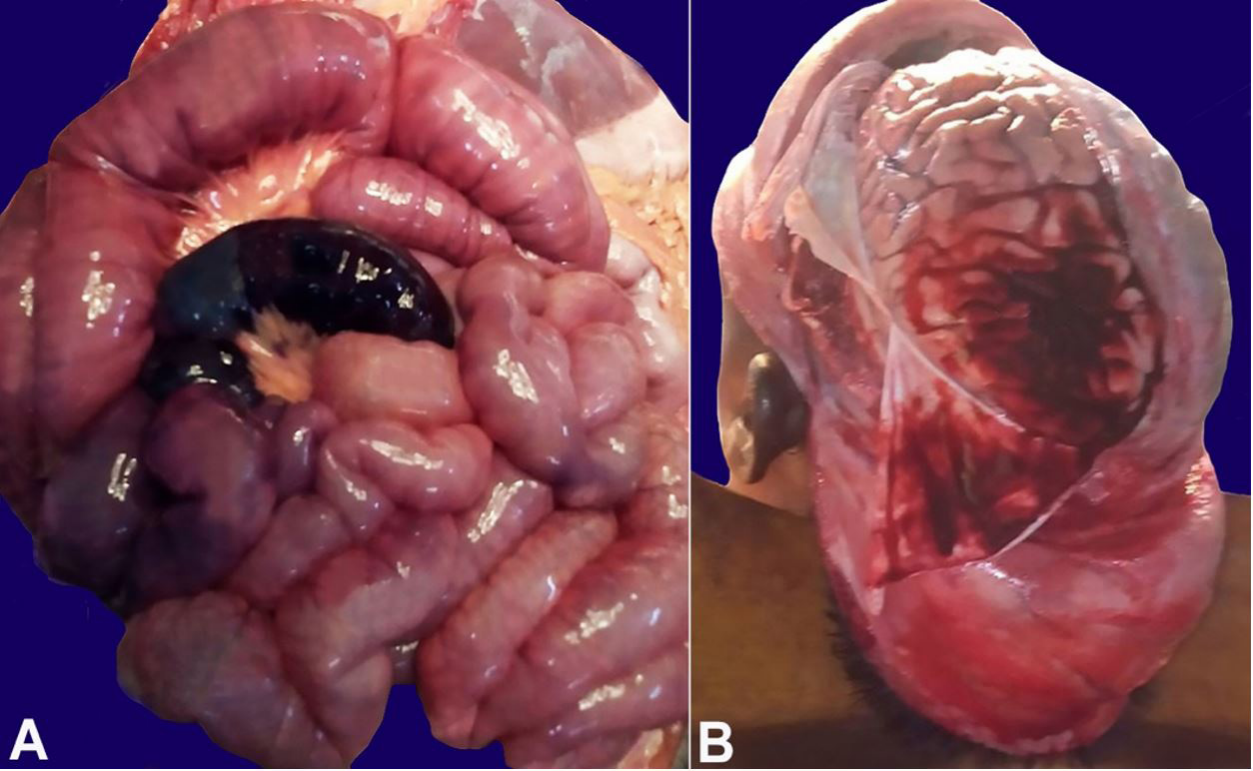
A – Gross picture of the small intestines showing marked congestion and a gangrenous segment; B – Large left subdural hemorrhage in left temporoparietal area.

An autopsy revealed the body of a eutrophic young male. Thin adhesions were seen between the lung and the parietal pleura on the left side. The lower lobes of both lungs were congested and oozed pink fluid. The peritoneal cavity contained minimal blood-stained fluid. The small intestine showed marked congestion with two gangrenous segments measuring 15 cm and 7 cm (Figure 1a). The stomach showed a hemorrhagic mucosal surface. Bilateral iliopsoas hematomas were seen, and a large left subdural hemorrhage (Figure 1b) was seen on opening the cranial cavity. Spleen was enlarged, weighing 350 g (reference range:120-200g), and showed congestion at the cut surface. No splenic infarcts were seen. The cut surface of the brain parenchyma, heart, kidneys, liver, adrenals, pancreas, urinary bladder, and testes were grossly unremarkable.

**Figure 2:**
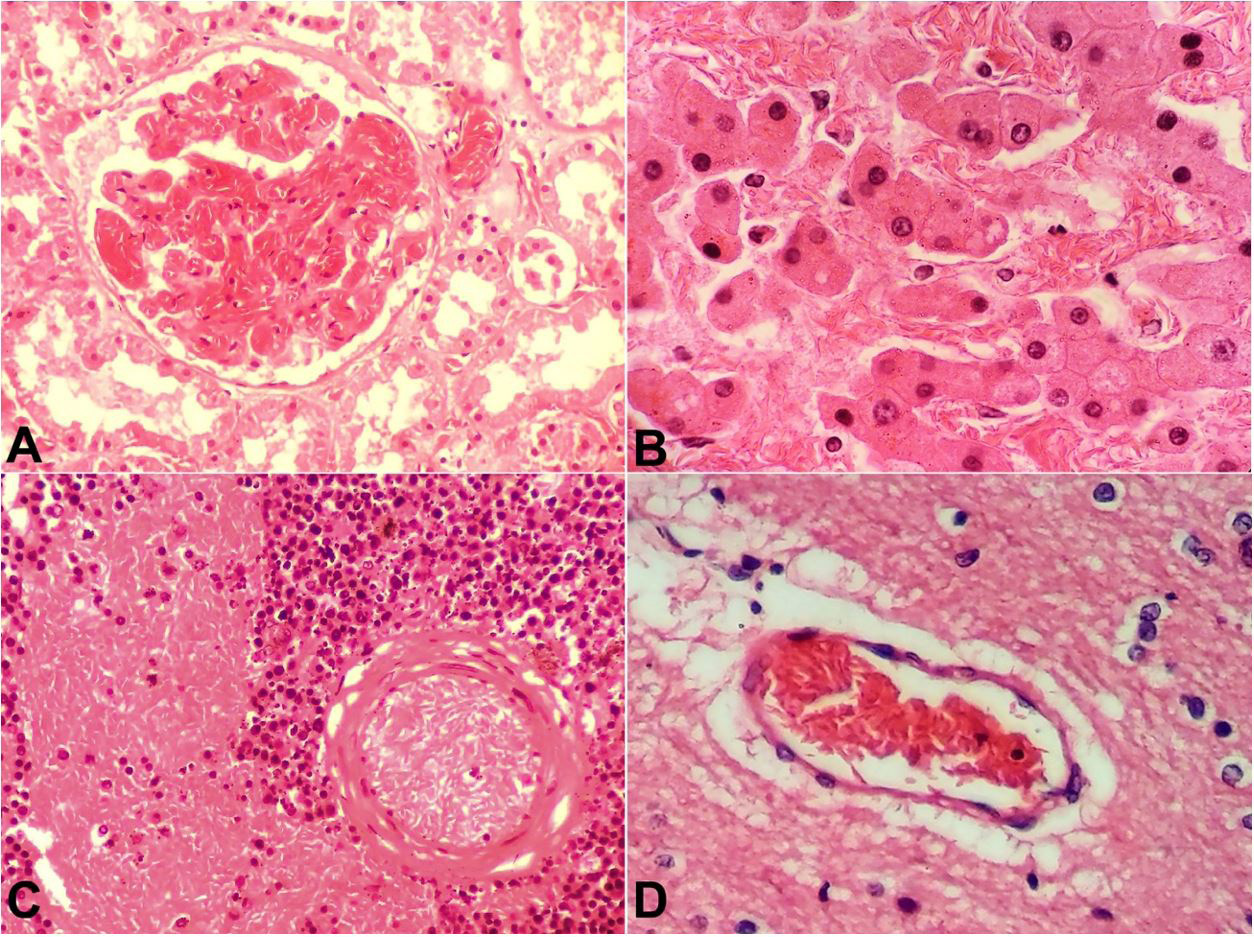
Photomicrographs of: A – kidney showing sickled red blood cells clogging glomerular capillaries (H&E, 400x); B – Liver showing hepatic sinusoids congested with sickled red cells and disarrangement of hepatic trabeculae. (H&E, 400x); C – spleen showing microvascular occlusion within the capillaries and congestion of red pulp by sickled red cells (H&E, 400x); D – Cerebral capillaries showing sickled red cells with perivascular space enlargement. (H&E, 400x).

On microscopy, acute congestion and microvascular occlusion by sickled red cells were seen in the glomerular capillaries, hepatic sinusoids, splenic red pulp, brain (figures 2a-d), pancreatic vasculature, adrenals, intestines, lymph nodes, and skeletal muscle. Pleural fibrosis was seen. Alveolar spaces of lower lobes showed congestion. Kidneys showed proximal tubular necrosis. Sections of psoas muscle showed myonecrosis. However, no myoglobin intratubular casts were seen. There was no histopathologic evidence of any inflammatory or infectious disease.

Postmortem high performance liquid chromatography (HPLC) performed on deceased blood revealed SCT (HbS 38.5%, HbA 58.2%, HbA2 2.4% and 0.9% HbF). A similar pattern was seen in his father’s blood. Death was attributed to multiorgan damage associated with massive intravascular sickling due to exertion-induced hypoxia-related to extreme physical activity and in an undiagnosed SCT case.

## DISCUSSION

Sickle cell trait, although believed to be an innocuous condition, is associated with certain clinical sequelae with increased frequency. These include (i) isosthenuria, (ii) renal hematuria, (iii) bacteriuria and pyelonephritis in pregnancy, and (iv) splenic infarction with high altitude hypoxia.^9^ Sickle cell hemoglobinopathy is common in India, but sudden death after physical exertion has never been reported.^3^^,^ 4 The Indian armed forces do not have a policy for screening recruits at the entry for hemoglobinopathies, nor is there a neonatal mass screening in India, unlike many developed nations like the United States and the UK.

Many authors have cited that other factors in SCT such as intensity of exercise, dehydration, associated viral illnesses, heat stress, and high altitude have a role in the trigger for collapse and sudden death. Of all these, the intensity of exercise appears to be the strongest incriminating factor.^6^^,^ 7 ^,^ 8 ^,^ 10 The deceased, in this report, had completed four months of military training and had already reported three episodes of exertional collapse, the final one being fatal. During the course of military training, the intensity of exercise is gradually increased. Maximal physical effort results in hypoxemia, hyperthermia and lactic acidosis within the muscles. This leads to red cell dehydration as they pass through the muscular hyperosmotic environment. This could be associated with a significantly higher risk of exertional rhabdomyolysis.The outcome is the concentration of the HbS, resulting in sickling in the microcirculation. 10 Low level of oxygenation in the cell is the prime physiologic cause of intravascular sickling in SCT patients. 11

Repeated sickling episodes may lead to local necrosis in the renal papillae, resulting in isosthenuria (the inability to concentrate urine) and consequent dehydration.^11^ Exertional rhabdomyolysis as a result of sickling could elevate the risks of glomerular and tubular damage caused by myoglobin toxicity, followed by acute renal failure.^12^

It also contributes to the release of proinflammatory cytokines and procoagulant factors.^13^ The cumulative effects of all these factors in the bloodstream may have led to the development of an irreversible ‘metabolic storm’ similar to this case.

At previous admissions, the presyncope in this young recruit was misconstrued to cardiac causes, and the extensive cardiac evaluation was normal. Heatstroke was another differential diagnosis. However, the area of the training center is located at a mean height of 3000 feet above sea level and does not experience temperatures beyond 22 degrees in the morning hours, even during the summer months. It also excludes high altitudes as an adjunct to hypoxemia.

In a large study of 139 cases of non-traumatic exercise-related deaths in recruits, the commonest cause of death cited were cardiac causes (59%) namely coronary artery anomalies, myocarditis, atherosclerotic cardiovascular disease, and cardiomyopathy. Exertional heat injury was contributory to death in one-third of all cases, and SCT was identified in 26(18.7%) of all cases.^14^ A retrospective review by Kark et al.^14^ concluded that the risk of unexplained sudden death in African recruits with sickle cell trait was 30 times higher than those without SCT. Most deaths had occurred during the initial months of training and were associated with physical activities involving maximal exertion.

SCT is diagnosed by ion-exchange HPLC. The peripheral blood and Hb values are usually normal in an SCT. Tests for sickling such as sodium metabisulfite tests can easily be performed in smaller laboratories. Till the advent of a neonatal screening program or screening for SCT at entry into the armed forces, if an individual has normal hematological values at the entry to the army, like in this case, it is impossible to diagnose a hemoglobinopathy.

## CONCLUSION

As demonstrated in this case and other reports in the literature, severe exertion can cause sickling and microvascular occlusion in an SCT also. A high index of suspicion and awareness to prevent a crisis is required. Until universal screening policy comes into force, especially in poor resource and developing nations with high sickle cell prevalence; this case serves to remind all sports physicians and doctors of the armed forces of the importance of screening a recruit who is unable to complete his scheduled physical training for the presence of SCT.
